# ASPIRE for quality: a new evidence-based tool to evaluate clinical service performance

**DOI:** 10.1186/s13104-016-2109-0

**Published:** 2016-06-13

**Authors:** Jeric Uy, Lucylynn Lizarondo, Alvin Atlas

**Affiliations:** International Centre for Allied Health Evidence, School of Health Sciences, University of South Australia, City East Campus, Adelaide, SA Australia

**Keywords:** Performance evaluation, Quality, Measurement, Allied health

## Abstract

**Background:**

Evaluation of clinical performance is important in allied health but without a structured approach, the measuring or monitoring of allied health performance poses a number of challenges. This highlights the need for an evidence-based evaluation tool to assist allied health practitioners in clinical performance evaluation.

**Methods:**

The ASPIRE framework was delivered to local health networks (LHN) in South Australia. Three sites participated in the pilot by providing a team to collaborate with the authors in organising and undertaking a performance evaluation. Evaluation of ASPIRE was conducted via self-administered questionnaire and a semi-structured interview with the evaluation team. Themes were identified from the responses taken from the questionnaire and interviews.

**Results:**

All practitioners found ASPIRE useful or very useful and claimed that it helped quite a lot or a lot in the process of undertaking performance evaluation. They all rated ASPIRE as excellent or very good in terms of its appropriateness to their department, ease of implementation and pace of delivery. The interview findings verified the results of the questionnaire and added richness to the evaluation.

**Conclusion:**

A pilot test of ASPIRE in allied health settings showed that users found ASPIRE easy to use and appropriate in addressing patient outcomes and improved their level of confidence and motivation to evaluate clinical performance. Issues arose in terms of time constraints and identifying suitable performance indicators. Future implementation of performance evaluations using the ASPIRE framework should take these issues in consideration to allow the tool to be refined and be relevant for use.

## Background

In healthcare, performance evaluation is intended to monitor, evaluate and communicate the extent to which various aspects of the health system meet their key objectives [1]. Allied health is a diverse and broad term covering multiple disciplines, providing not just direct patient or therapy services, but also involving diagnostic or technical services and education [2]. Such diversity creates a challenging scenario in regards to performance evaluation, as the delivery of allied health care is unique to each discipline and will present with different performance needs that will therefore require different evaluation approaches [3]. As allied health professionals take on a more advanced and extended scope of practice [4–6], the evaluation of clinical service performance is becoming essential in order to identify strengths and weaknesses to improve future performance [7], and to ensure that services are targeted [8] and cost effective [9]. The selection and implementation of an effective clinical service assessment strategy is often challenging for allied health practitioners, the individual disciplines have different objectives and purposes, varied ways of operation, stakeholders, outcomes and quality measures. As such, there is no one-size-fits-all approach or one agreed approach for performance evaluation that can be recommended to all allied health care settings [3]. This presents a clear need for an individualised and tailored evidence-based evaluation tool to assist allied health practitioners in clinical performance evaluation.

Allied health clinical performance evaluation should be underpinned by processes that are based on research and with an understanding of the perspectives of different stakeholders (i.e. allied health practitioners, managers/directors, consumers). It should be reinforced by a long-term vision to improve overall health outcomes, health service delivery, workforce performance and healthcare utilisation and cost.

This paper describes the development of ASPIRE, an evidence-based tool to evaluate clinical service performance and its pilot and evaluation in the short term. ASPIRE was developed to address the challenges experienced by allied health practitioners and provide a structured guidance in undertaking the process of evaluation, with the ultimate aim of improving the quality of allied health services.

## Methods

### Ethics approval

Approval for the survey process and pilot evaluation was obtained from the University of South Australia Human Research Ethics Committee and South Australia Health Human Research Ethics Committee.

### Development of ASPIRE

ASPIRE was designed following a review of the literature on clinical performance evaluation [3] and a survey involving allied health managers from the five local health networks (LHNs) in South Australia, namely Central Adelaide LHN, Northern Adelaide LHN, Southern Adelaide LHN, Women’s and Children’s Health Network and Country Health South Australia LHN. The LHNs manage the delivery of public hospital services and other community-based health services as determined by the State Government. They comprise single or groups of public hospitals which have a geographical or functional connection. The LHNs are accountable to the state government for performance management and planning.

Based on the review of the literature, underpinning an effective performance evaluation system are core processes or elements that include prioritisation of clinical area for evaluation, upfront articulation of goals, careful identification of performance measures, mapping of measures to information sources and analysis of performance data and reporting of results [9]. A careful examination of barriers to performance evaluation and the subsequent tailoring of strategies to overcome these barriers are important to achieve the aims of evaluation. The survey, on the other hand, captured a local snapshot of current practice in performance evaluation in South Australian allied health LHNs. Results have shown that local practices are generally based on widely accepted tools and principles. While all survey respondents valued the role of performance evaluation, the majority reported various challenges associated with the process. These include lack of time, limited understanding of the process and lack of a standard framework to undertake performance evaluation. Respondents believed that training on how to conduct performance evaluation and a standardised evaluation framework to guide and support evaluators would be useful. To facilitate timely and efficient evaluation, support from an external experienced evaluator or allocating a position dedicated to performance evaluation were identified as potential strategies.

Integration of the review findings and survey results led to the development of ASPIRE, an evidence-based framework that provides allied health practitioners with a structured process (as shown in Table 1) as well as a toolkit (Appendix 1) to facilitate performance evaluation. The ASPIRE framework captures the core elements of performance evaluation and recognises the barriers or challenges associated with the process. It utilises a collaborative approach between allied health practitioners and experienced researchers who have extensive evaluation skills needed for the proposed evaluation model. ASPIRE divides the core tasks between researchers and allied health practitioners from the health site, as outlined in Table 1. The researchers provide strong initial support and guidance which gradually reduces to enable practitioners to establish and maintain independence and promote a sense of ownership of the performance evaluation system.

**Table 1:** ASPIRE for quality framework

**A**rea for evaluation*	The evaluation team from the allied health site identifies and prioritises the clinical area for performance evaluation
**S**et goals*	Based on the identified clinical area, the evaluation team sets the goals for performance evaluation
**P**erformance indicators**	The evaluation team, assisted by experienced researchers, identifies performance measures or indicators
**I**nformation sources*	The evaluation team maps the performance measures to information sources
**R**eport results**	The researchers and evaluation team collaboratively analyses the results and report to stakeholders
**E**valuate**	The researchers and evaluation team collaboratively evaluates the performance evaluation system

* Tasks are responsibilities of allied health practitioners

** Tasks are shared responsibilities of researchers and practitioners

### Pilot evaluation using the ASPIRE framework

Following a recommendation by the Chief Allied Health Advisor, Allied Health and Scientific Office of the Department of Health, South Australia, three allied health sites volunteered to join the pilot, which was conducted from January to May 2014. The ASPIRE framework was delivered by two experienced researchers (LL, AA) with extensive expertise in health service evaluation and epidemiology and in providing evaluation training. Prior to the implementation of the clinical performance evaluation pilot using the ASPIRE framework, each site was instructed to organise an evaluation team who worked closely with the researchers in undertaking performance evaluation. The three-person team consisted of the manager and/or senior allied health staff.

Allied health directors representing the five LHNs in South Australia were approached via email to invite allied health managers to participate in the pilot implementation. The ASPIRE framework and toolkit was offered as an incentive to encourage participation. As time and funding did not allow for a large scale evaluation, recruitment was limited only to three sites that represent a metropolitan rehabilitation hospital, a metropolitan acute tertiary hospital and a regional general hospital. Written informed consent was obtained from all allied health professionals who volunteered to participate.

### Evaluation of ASPIRE

The evaluation process entailed a self-administered questionnaire and a semi-structured interview with members of the allied health evaluation team.

At the end of performance evaluation using ASPIRE; members of the allied health evaluation team completed a brief online questionnaire asking for comments and views about its usefulness, acceptability and appropriateness to allied health clinical practice and the extent to which it met their expectations. Results were collated and the percentage of respondents providing a specific response was calculated for each question. The online questionnaire allowed for free comments, which were collated. Themes were identified by two investigators (LL, AA) and examples extracted to illustrate reactions and perspectives about ASPIRE.

Semi-structured interviews, which lasted for about an hour, were also undertaken to validate the results of the questionnaire and explore participants’ views in more depth. The following broad questions were used as a guide during the interview:

- What are your perceptions regarding ASPIRE as a framework for your routine performance evaluation in your department?
- What are your impressions of how well your team embraced the ASPIRE to facilitate performance evaluation?
- What are your perceptions of what works well and what does not work well in the ASPIRE framework?
- What difference did ASPIRE make in the conduct of your performance evaluation?

Using content analysis, two investigators (LL, AA) independently coded the interviews and then collaborated to distil the codes into content-related categories and themes. Coding was undertaken manually, highlighting different categories with different colours. A summary of the key themes was provided to all participants to verify if they were congruent with their responses. Comments that illustrated the emerging themes were selected.

## Results

Three sites participated in the pilot implementation and short-term evaluation of the ASPIRE framework. A summary of the performance evaluation areas, goals and team members are presented in Table 2.

**Table 2:** Summary of the performance evaluation process from the three sites

	Area for evaluation	Goal	Evaluation team
*Site 1*–*metropolitan rehabilitation hospital*	Rehabilitation following unilateral below knee and above knee amputation	To examine practice compliance against established clinical guidelines for amputation in order to stimulate improvements in allied health services, which could potentially improve patients’ functional outcomes and decrease their length of stay in rehabilitation	Physiotherapist, occupational therapist and a social worker
*Site 2*–*metropolitan acute tertiary hospital*	Depression or mood disturbance following a stroke	To determine the impact of implementing a structured mood tool in identifying patients who are likely to be depressed or are experiencing mood disturbance following a stroke episode. The mood tool complies with the national stroke guidelines recommendation of a structured and psychometrically sound instrument to detect early mood changes (i.e. depression) and therefore facilitate timely referrals to psychological assessment and treatment	3 Social workers
*Site 3*–*regional general hospital*	Foot screening in diabetes care	To examine compliance of current practice in foot screening against the national evidence-based guideline for the prevention, identification and management of foot complications in diabetes	2 Podiatrists

Six (i.e. two from each site) of the eight practitioners completed the questionnaire and agreed to be interviewed.

All practitioners found ASPIRE useful or very useful and claimed that it helped quite a lot or a lot in the process of undertaking performance evaluation. They all rated ASPIRE as excellent or very good in terms of its appropriateness to their department, ease of implementation and pace of delivery. Many highlighted the value of ASPIRE in addressing issues which were considered problematic in the past; others appreciated the guidance provided by the framework and the support from researchers. They commented that the combination of skills between the staff members and the researchers provided not just the needed oversight but also the needed confidence to maintain the momentum of the project going. The practitioners often compared their previous evaluation process with that of ASPIRE and commented that ASPIRE tends to be more patient-centred. They also appreciated that ASPIRE was based on guidelines for patient care rather than funding related measures.

Sixty-seven percent (4/6) said ASPIRE performed above the department’s expectations and 33 % (2/6) expressed that it was far above their expectations. All practitioners reported that their level of confidence and motivation to undertake performance evaluation moderately or significantly improved. Eighty-three percent (5/6) evaluated the support received from researchers as excellent and 17 % (1/6) said it was good. Practitioners reported they are likely or very likely to use ASPIRE in their next round of performance evaluation.

The views and experiences of allied health practitioners regarding the use of ASPIRE for performance evaluation were classified into: strengths of the framework, challenges associated with performance evaluation using ASPIRE and refinements to the ASPIRE framework.

### Strengths of the ASPIRE framework

The participants agreed that working together with experienced researchers is an effective strategy to encourage allied health to evaluate their clinical performance. They found the framework useful in providing them a structure or a step-by-step guide in undertaking a performance evaluation. The participants felt that partnership between allied health evaluators and researchers is a blending of expertise, with researchers facilitating the research component (e.g. development of data abstraction forms, analysis of data) while clinicians provide an understanding of the work environment and clinical context.

*‘One thing I found daunting is taking on the task of developing a whole structure and how it’s going to happen, what’s going to be meaningful…but you helped us with those things. There was an organised structure…it was very good. Being involved in the process gave us a sense of ownership.’*

One of the participants commented:

*‘It saved us quite a bit of time. It was a different way of thinking. You simplified it and it didn’t seem to be cumbersome because you can be frightened about the evaluation process but you made us feel that we can do this…it’s that encouragement that we got because it didn’t seem like a complex process, and you guide us through.’*

One of the sites recognised the value of including process measures in the evaluation and how these can be linked to outcomes.

*‘Going through those process measures is a good way of making sure that we do improve those things, which could potentially affect the outcome.’*

One of the sites also noted that going through the clinical guidelines as part of the process of identifying key performance indicators was a useful exercise for reflective practice. The participants recognised the value of evidence-based recommendations; however, they are not always up-to-date with scientific information.

*‘Being made aware of the clinical guidelines was very useful because we’re not always aware of the breadth of things that are out there….which makes you think, ahhh we’re doing these but maybe we don’t.’*

All participants agreed that undertaking performance evaluation using ASPIRE created an environment for change and challenged them to think of more ways to improve the quality of their services. It also offered them an opportunity to reflect on their own clinical performance and discuss as a team potential strategies to correct or improve practice behaviour. One of the participants commented:

*‘This evaluation identified that many of the assessments that we do are not properly or adequately documented. We know that a lot of us do this but we don’t necessarily write them in the notes, which in itself is a legal issue. We need to revisit our documentation and because we have this report…we can say, look…this is what’s happening and we have to do something about it.’*

All sites commented they feel more confident undertaking performance evaluation on their own in the future. One of the participants said,

*‘Now I can say that I can replicate the same process next time. Even just the setting up of excel for data audit is something I would have never done that meticulously before. Or even the identification of performance indicators…it became so much easier when we were given access to best practice guidelines and then as a team we identified which ones are likely to impact on length of stay.’*

### Challenges associated with performance evaluation using ASPIRE framework

The challenges raised by the participants were not specific to the use of ASPIRE but rather common to any process of performance evaluation. One of the participants reported that identification of process indicators that are relevant to their outcome of interest was quite challenging, particularly if there are several process recommendations in best practice clinical guidelines.

*‘I found it difficult to know which of those processes from the guidelines would affect the outcomes.’*

Time to collect or abstract data from clinical case records was also a concern for some participants.

*‘The resources available, personnel to abstract the data, on top of all the work that we need to do can be quite challenging.’*

*Refinements to the ASPIRE framework to facilitate effective and sustainable uptake in allied health.*

Overall, the participants were positive about ASPIRE and felt that performance evaluation using a framework was a worthwhile experience. However, they believed that there are still opportunities for improvement which could increase its effectiveness. The most telling comments came from participants who felt that the evaluation process could have been more effective if there was longer time spent on planning the evaluation.

*‘Longer planning time especially when developing the data abstraction sheet to develop a common understanding of what should be abstracted.’*

Participants from the regional site suggested that a face-to-face consultation, rather than a teleconference, is beneficial particularly during the early stages of planning.

*‘Face*-*to*-*face contact and a visit to the site by the researchers during the planning process, rather than a teleconference, would be preferred.’*

Some participants felt that distilling performance indicators from evidence-based clinical guidelines could have been an easier process if a wider team was involved.

*‘The idea of having a wider team to discuss the guidelines to identify the indicators would be helpful.’*

## Discussion

Routine clinical performance evaluation is an integral component of health care quality and is a critical tool to promote improved health service delivery [10]. There is anecdotal evidence to show that allied health practitioners, while acknowledging the importance of performance evaluation, lack the confidence and feel unprepared for this work. This is not surprising given that performance evaluation raises several challenges for practitioners, particularly around selection of performance measures and implementation of an effective evaluation strategy [11]. ASPIRE was developed to address these barriers and challenges to performance evaluation. The pilot in three different allied health sites showed that ASPIRE was well-received and highly valued by the practitioners. Especially encouraging was the finding that the evaluation teams were keen to use ASPIRE for future evaluations.

The ASPIRE framework takes a practical approach, attempting to tackle the difficulties associated with performance evaluation by adapting a partnership model between experienced researcher-evaluators and allied health practitioners, at least during the initial evaluation. A ‘Guide to Evaluation in Health Research’ released by the Canadian Health Institute of Health Research, reported that ‘*research skills are required to ensure that such evaluations* (*which inform not only decisions about continuing or spreading an innovation, but also whether to discontinue current services, or change established processes*) *are well designed, implemented and interpreted’* [12]. Mainz (2003) argued that quality of care researchers with clinical epidemiological expertise can help ensure methodological integrity of the clinical indicators and a valid approach to data collection and analysis. In partnering with experienced researchers, ASPIRE brings together a useful combination of contextual knowledge and technical evaluation skills which are required to facilitate appropriate use of results and therefore achieve the best outcomes for the health service department or organisation. ASPIRE also aims to build the evaluation skills of practitioners to allow them to conduct evaluation on their own, in a more effective and efficient way. As a result it fits particularly well for practitioners who feel uncertain of the process and lack the confidence and motivation to undertake a seemingly daunting practice.

A number of evaluation framework for healthcare is available and in fact, became the foundation for ASPIRE [6, 7, 9, 11–13, 15]. ASPIRE expanded what already exists and recognised local barriers to evaluation and as a result, offers a practical, step-by-step process and a toolkit that allied health practitioners can use to facilitate the process of performance evaluation. Measurement of clinical performance in allied health in South Australia is characterised by a lack of standardised framework to guide practitioners and as a result, a lot of variability exists in current practice. Evaluating clinical performance is not a simple process and can sometimes generate massive amounts of data which often overwhelm practitioners [10, 12]. By using a simple and practical approach to performance evaluation, ASPIRE encourages allied health practitioners to take a small step in performance evaluation rather than attempting to implement a massive, unrealistic performance measurement program. By starting with a very focused, realistic and attainable performance evaluation activity, the chance for successful implementation is likely to increase, which can then set the stage for the later development of more complex performance evaluation. Buy-in is also likely to increase when an evaluation team can demonstrate a history of successful initiative [14].

Motivation from both managers and individual practitioners to participate in a clinical performance evaluation process is a major challenge to implementation [15]. Often staff members are sceptical about the usefulness and value of performance evaluation [14, 15]. Participants in the ASPIRE pilot reported that the evaluation process was a worthwhile experience and indicated that ASPIRE was a useful and appropriate tool for clinical performance evaluation. Furthermore, participants also reported that ASPIRE improved their level of confidence and motivation to conduct performance evaluation.

### While the findings are encouraging, it is important to consider limitations

Clearly, more rigorous, independent evaluation is required before the findings can be considered conclusive. What it does suggest however, is that ASPIRE is an approach that will provide a basis for standardisation of the performance evaluation process and that it addresses an area that has been identified by allied health practitioners as challenging. This study also contributes to the existing body of knowledge by addressing the gap that currently exists in allied health performance evaluation methods and measures. A key outcome of this research is the development of an evidence-based framework that can encourage implementation of a process known to improve the quality of allied health care services.

While this research has served to provide guidance to practitioners, future research is needed to further explore the value and usefulness of ASPIRE for specific allied health disciplines in different settings. It would also be worthwhile to compare the outcomes of performance evaluation between those with access to ASPIRE training and toolkit to those without, or perhaps compare ASPIRE with a different evaluation model. In addition, the true value of performance evaluation lies in its ability to show that improvements in health care are a result of the evaluation and that the health system is making data-driven decisions. As such, future studies should evaluate the impact of performance evaluation using ASPIRE on overall health outcomes, health service delivery, allied health workforce and healthcare utilisation and cost.

Exploring the use of information technology to better access and share data would facilitate the ease of use of ASPIRE in the clinical setting. The availability of internet access and portable computer devices would also allow health workers to retrieve the information needed to map out a specified performance measure. The feasibility of designing and developing a software application based on ASPIRE to be used for smartphones and portable computing tablets should also be considered.

Finally, a fundamental component of health service delivery is the recognition of the importance of consumer engagement in healthcare decisions [16]. It is therefore vital that mechanisms are in place to actively engage with consumers when organising clinical performance evaluation. Future studies should also investigate strategies that will ensure consumer representation in the process of evaluation.

## Conclusion

The evaluation of clinical service performance is an essential task in establishing the effectiveness and value of interventions. It also provides important insight to the gaps in service delivery and identifies potential opportunities for improvement and innovation. A pilot use of ASPIRE in allied health settings showed that a collaboration between researchers and clinicians was useful in evaluating clinical performance. Users found ASPIRE as easy to use and appropriate in addressing patient outcomes and improved their level of confidence and motivation to evaluate clinical performance. Issues arose in terms of time constraints and identifying suitable performance indicators. Future implementation of clinical performance evaluation using the ASPIRE framework should take these issues in consideration to allow the tool to be refined and be relevant for use and determine if the tool had a positive effect on the delivery of care services.

## Appendix: ASPIRE performance evaluation tools

*Choose the clinical area you want to evaluate* Undertaking performance evaluation can be a laborious and time-consuming process and for it to be meaningful, carefully selecting a clinical area for evaluation is very important [17–23].

**Table:** Summary of clinical performance evaluation system

Clinical performance area for evaluation	Example: Rehabilitation following unilateral below knee and above knee amputation		
Goals *Describe what the department/organisation aims to achieve; should be SMART (Specific, Measurable, Attainable, Realistic, Time bound)*	Example Examine compliance with guidelines; improve services and decrease the length of stay		
Performance Measures *A performance measure or indicator is used to assess a particular health care structure, process or an outcome. It is based on standards of care, which can be evidence*-*based or, in the absence of scientific evidence, determined by an expert panel of health practitioners based on their experience.*	*Structure* Evaluate the means and resources used by the health system to deliver services Examples Existence of a locally agreed, amputee-specific outcome measure for gait Existence of a protocol for checking the residual limb before, during and after treatment	*Process* Examine the interaction between health practitioners and patients; assess what the health practitioner did for the patient and how well it was done Examples Percentage of patients whose gait was evaluated using a validated outcome measure Percentage of patients provided with falls education	*Outcome* Examine the change in patients’ health status which can be attributed to the effectiveness of the treatment. Examples Average length of stay (for below knee amputees and for above knee amputees) Average % change in FIM (Functional Independence Measure) score
